# Uncover miRNA-Disease Association by Exploiting Global Network Similarity

**DOI:** 10.1371/journal.pone.0166509

**Published:** 2016-12-01

**Authors:** Min Chen, Xingguo Lu, Bo Liao, Zejun Li, Lijun Cai, Changlong Gu

**Affiliations:** 1 School of Information Science and Engineering, Hunan University, Changsha, China; 2 School of Computer and Information Science, Hunan Institute of Technology, Hengyang, China; University of Texas MD Anderson Cancer Center, UNITED STATES

## Abstract

Identification of miRNA-disease association is a fundamental challenge in human health clinic. However, the known miRNA-disease associations are rare and experimental verification methods are expensive and time-consuming. Therefore, there is a strong incentive to develop computational methods. In this paper, we calculate the similarity score for each miRNAs pair by integrating miRNA functional similarity and miRNA family information. We use the disease phenotype similarity data to construct the disease similarity network. Then we introduce a new miRNA-disease association prediction method (NETwork Group Similarity, NetGS) to explore the global network similarity, capturing the relationship between the disease and other diseases, the similarity between the potential disease-related miRNA and other miRNAs. Finally based on the consistency of diffusion profiles we get the miRNA-disease association scores. NetGS is tested by the leave-one-out cross validation and achieves an AUC value of 0.8450, which improves the prediction accuracy. NetGS can also be applied to solve the new miRNA-disease association and obtain reliable accuracy. Moreover, we use NetGS to predict new causing miRNAs of three cancers including breast cancer, lung cancer and Hepatocellular cancer. And the top predictions have been confirmed in the online databases. The encouraging results indicate that NetGS might play an essential role for future scientific research.

## 1. Introduction

MicroRNAs (miRNAs) are a type of short non-codingRNAs which strongly affect cellular functions including ceil differentiation, proliferation and apoptosis [1–3]. In recent years, increasing evidences show that miRNAs play important roles in the development and progression of human diseases [4–8]. Uncovering disease-related miRNAs has significant impact to formulate individualized treatment regimes [9].So far, several experiment methods, such as microarray profiling and qPT-PCR, have been proven successfully. However, identifying microRNA-disease associations by the existing techniques is expensive and time-consuming [10]. As a result, it is necessary to develop computational methods to uncover miRNA-disease associations [11]. The computational methods can select most likely disease-related miRNA for future analysis which decreases the number of experiments and help to understand the miRNA functions. At present, the biggest challenge of prediction task is the rarity of known miRNA-disease associations [12].

To predict the miRNA-disease associations, some important conclusions and computational approaches have been proposed. Lu et al. manually collected miRNA–disease associations from published literatures and constructed the database HMDD v2.0 [13].They further analyzed the human microRNA–disease association data and obtained several important patterns between miRNAs and diseases. One of the most important conclusions in their paper is that phenotypically similar diseases tend to be associated with functionally related miRNAs, which has been treated as the basic assumption of many current disease-related miRNA prediction methods. Based on this assumption, Jiang et al. first constructed a miRNA functional network based on hypergeometric and further inferred miRNA-disease associations using phenotype similarity information [14]. However, their method only considered the direct neighbor information of miRNAs in the network and strongly relied on the predicted miRNA-target data, in which some false positive and false negative results existed. Xu et al. proposed an approach based on the topological feature of the target-deregulated network [15]. Some negative samples were used in their model. However, as we known, negative miRNA-disease associations can not be confirmed by current technique. Chen et al. predicted miRNA-disease association method RWRMDA based on the miRNA-miRNA similarity network, which was constructed by the similarity of miRNA-related diseases [16]. Unfortunately, RWRMDA is not applicable to orphan disease with no known related-miRNA information. Based on the functional link between miRNA targets and disease genes in the protein-protein interaction network, shi et al. proposed a computational framework to identify miRNA-disease associations. Although good prediction results have been achieved, their method strongly relies on the known disease–gene interactions and miRNA–target associations [17]. Chen and Zhang proposed three inference methods to predict miRNA-disease associations based on different similarity measure strategies [18]. Chen and Yan also introduced a semi-supervised learning method for miRNA-disease association prediction [19]. However, the prediction accuracies of these two methods still need to be improved.

Considering the problems mentioned above, we first introduce miRNA family information to improve the miRNA similarity and propose a new miRNA-disease association prediction method (NetGS) to explore the global network similarity. In the prediction procession, graph laplacian scores are used to calculate the global similarity of miRNAs and diseases. The idea of diffusion profile consistency is used to compute the miRNA-disease association score. Cross-validation and case studies about three kinds of cancers have fully demonstrated that NetGS is superior to existing methods. It may be great significance for the future research in this area.

## 2. Materials

### 2.1 The human miRNA-disease association data

We downloaded miRNA-disease associations from [14] and took it as the gold standard dataset in the cross validation (Please see the S1 File). It includes 270 high-quality experimentally verified miRNA-disease associations. After removing 19 miRNA which can’t be found in [20], 242 distinct experimentally confirmed miRNA-disease associations were obtained, including 99 miRNA and 51 diseases.

Besides, to validate that our method is not sensitive to the dataset, another miRNA-disease association dataset was downloaded from supplementary material of [20].This dataset contains 1616 experimentally verified human miRNA-disease associations (obtained from HMDD v2.0 2014). After merging the records of different miRNA copies and unifying the names of miRNAs and diseases, 1395 miRNA-disease associations were obtained, including 271 miRNAs and 137 diseases. In the following, we call this dataset as the second miRNA-disease association dataset.

### 2.2 miRNA functional similarity data

The miRNA-miRNA functional similarity scores were downloaded from [20]. In this dataset, the miRNA functional score for each pair was calculated based on the observation that functionally similar genes are often associated with similar diseases. The miRNA functional similarity data have been successfully used in [16, 18]. Here, we denote matrix SM^f^ as the adjacency matrix of miRNAs, where SM^f^ (i, j) in row i column j is the functional score between miRNAs i and j.

### 2.3 The miRNA family information data

We collected miRNA family information from the latest miRBase19.As we know that miRNAs in the same family likely share common set of mRNA target. As a result, they are more likely associated with similar diseases [21].Here, Let matrix SM^fam^ as the adjacency matrix of miRNAs, whereSM^fam^(i, j) in row i column j is set to 1 when miRNA i and miRNA j are in the same family.

### 2.4 The disease phenotype similarity data

Phenotype similarities were downloaded from the literatures [22]. In the article authors computed the phenotype similarity scores by text mining of phenotype descriptions in the OMIM database [23]. We denote matrix SD^p^ as the adjacency matrix of disease, where SD^p^ (i, j) in row i column j is the phenotype similarity between disease i and j. This data has been successfully used in disease-gene association prediction [24–26].

## 3. Methods

### 3.1 miRNA similarity network and disease similarity network

Before constructing the miRNA similarity network, we calculate the similarity score for each pair. The computation formula is as follows.

$$SM\left(i,j\right)=SM^{f}\left(i,j\right)*\left(1+SM^{fam}\left(i,j\right)\right)$$ (1)

SM^f^ is the miRNA functional similarity matrix; SM^fam^ is the miRNA family matrix. As we known, miRNAs in the same family are more similar. So, in the equation, we give higher score for miRNA pairs which are in the same family (SM^fam^ (i, j) = 1).

Here, we use all known breast cancer-associated miRNAs to illustrate the better performance of the scores introduced. When the miRNA family matrix is added to calculate the similarity score, these causing miRNAs have larger probabilities to be chosen during the random walk. In addition, a miRNA network is also constructed and visualized in the supporting information S2 File.

Based on the miRNA similarities, miRNA network is constructed. Given the miRNA similarity network G_m_ = <V_m_, E_m_>where V_m_ = {m_1_,m_2_,…,m_n_} is the set of miRNAs in the network, E_m_ is the set of interactions. If the similarity score of miRNA i and j is more than zero, the vertices m_i_ and m_j_ are linked by an edge in the network. The weight of this edge is the corresponding miRNA similarity score.

The disease similarity network is constructed based on the disease phenotype similarity data. Given the disease similarity network G_d_ = <V_d_, E_d_>where V_d_ = {d_1_,d_2_,…,d_n_} is the set of diseases in the network, E_d_ is the set of interactions. If the similarity score of disease i and j is more than zero, the vertices d_i_ and d_j_ are linked by an edge in the network. The weight of this edge is the corresponding disease similarity score.

### 3.2 The global similarity about the query disease and potential disease-related miRNA

To fully utilize the global network information, we compute the global relevance score between the query disease and other diseases in the disease similarity network. Here we take laplacian scores to exploit the modular information in the network. Laplacian scores have been successfully used in [18,27].Let the binary vector d = {d_1_,d_2_,…,d_n_} denotes the initial vector of the query disease i, where d_i_ is 1 and other elements are 0. $\tilde{d}$ denotes the final vector in which global similarity scores are stored. The computation formula of laplacian scores is as follows [28].

$$\min_{\tilde{d}}{\sum_{i,j}{\bar{SD}}_{i,j}\left({\tilde{d}}_{i}-{\tilde{d}}_{j}\right)}^{2}+\frac{1-\alpha }{\alpha }{\sum_{i}\left({\tilde{d}}_{i}-d_{i}\right)}^{2}$$ (2)

In Eq (2), ${\bar{SD}}_{}$ is the column-normalized matrix of SD. The first term is a smoothness penalty, we assume that the connected disease to get similar scores. The second term ensures the consistency with the query disease. Parameter *α*∈(0,1) balances the contributions from the two penalties. The close solution of Eq (2) is as follows.

$$\tilde{d}=\left(1-\alpha \right){\left(I-\alpha \bar{SD}\right)}^{-1}d$$ (3)

The global similarity between the potential disease-related miRNA and other miRNAs in the network is calculated in the similar way. Let the binary vector m = {m_1_,m_2_,…,m_n_} denotes the initial vector of the potential disease-related miRNA j, where m_j_ is 1 and other elements are 0. $\tilde{m}$ denotes the final vector in which global similarity scores are stored. The laplacian scores can be derived from the following formula.

$$\min_{\tilde{m}}{\sum_{i,j}{\bar{SM}}_{i,j}\left({\tilde{m}}_{i}-{\tilde{m}}_{j}\right)}^{2}+\frac{1-\beta }{\beta }{\sum \left({\tilde{m}}_{i}-m_{i}\right)}^{2}$$ (4)

The close solution of the above optimization equation is as follows.

$$\tilde{m}=(1-\beta ){\left(I-\beta \bar{SM}\right)}^{-1}m$$ (5)

### 3.3 Computing miRNA-disease association score based on the consistency of diffusion profiles

The diffusion profile of a disease is defined as the stationary distribution of all other candidate miRNAs in the miRNA similarity network under a random walk with start where global similarities between diseases are incorporated. The diffusion profile of a miRNA is obtained by smooth the information from the query miRNA to the whole network. Actually, we have already got the diffusion profile of a miRNA in 3.2. The diffusion profile of the potential disease-related miRNA is $\tilde{m}$. The idea of diffusion profile consistency has been successfully used in prioritization of candidate disease genes [25].The specific calculation process of disease diffusion profile is as follows.

First, we denote D_0_ as the initial vector of the query disease, in which equal probability is assigned to the nodes representing the causing miRNAs of the query disease, with the sum equal to 1.The diffusion profile of the query disease can be obtained by the random walk with restart algorithm. The rule is defined as following:

$$D_{t+1}=\left(1-r\right)\overset{——}{SM}D_{t}+rD_{0}$$ (6)

$\overset{——}{SM}$ is the column-normalize matrix of miRNA similarity matrix SM. *D*_*t*_ is the probability distribution vector at step t. the probability distribution in the vector can be stable after certain steps. We designate the stable probability distribution vector ${\tilde{D}}_{\infty }$ as the diffusion profile of the query disease based on the miRNA similarity network.

Considering the assumption that similar disease associated with functionally related miRNAs, the initial probability vector of the query disease is optimized by introducing initial distribution of similar diseases. The computation formula is as follows.

$${\tilde{D}}_{0}=D_{0}+\lambda \sum_{i=1}^{n}sim\left(d_{1},d_{i}\right)\cdot D_{0}^{i}$$ (7)

${\tilde{D}}_{0}$ denotes the optimized vector. Then the diffusion profile of the query disease can be calculated by the following equation.

$${\tilde{D}}_{t+1}=\left(1-r\right)SM_{norm}{\tilde{D}}_{t}+r{\tilde{D}}_{0}$$ (8)

After getting the diffusion profiles of the query disease and the potential disease-related miRNA, we can get the miRNA-disease association score F by calculating the Pearson correction of the diffusion profiles.

## 4 Results

### 4.1 Leave-one-out cross validation and evaluation criteria

To evaluate the performance of our method, Leave-one-out cross validation (LOOCV) was implemented. Before the experiment, we examined the gold standard dataset and removed some highly similar miRNA-disease associations. Because it is important to make sure that the test case is not found in the training dataset. Actually, now, there is no standard to measure the similarity of the two miRNA-disease associations. So, a simple method was given here. If two miRNAs share the same disease and the similarity of these two miRNAs is higher or equal to 0.9, we think these two miRNA-disease association is highly similar. In this method, we didn’t consider the situation that two diseases share the same miRNA. It is because the similarities of most disease pairs are lower or equal to 0.2. We think the associations in this situation are not similar. After implementing the operation described above on the gold standard dataset, 225 known miRNA-disease associations were obtained (including 99 miRNAs and 51 diseases).LOOCV was implemented on these miRNA-disease associations. We left out each known miRNA-disease association in turn as test case and further evaluated how well this association was ranked to the candidate cases. Here, all unconfirmed associations were regarded as candidate samples and the rest known associations were regarded as the training samples. The receiver operating characteristic (ROC) curve and AUC value were employed to evaluate the prediction performance. The method (NetGS) proposed here achieved an AUC value of 0.8450. It indicates that our method can recover known experimentally-verified miRNA-disease associations and have the potential to infer new miRNA-disease associations.

### 4.2 Association prediction for orphan disease and new miRNA

**Fig 1:**
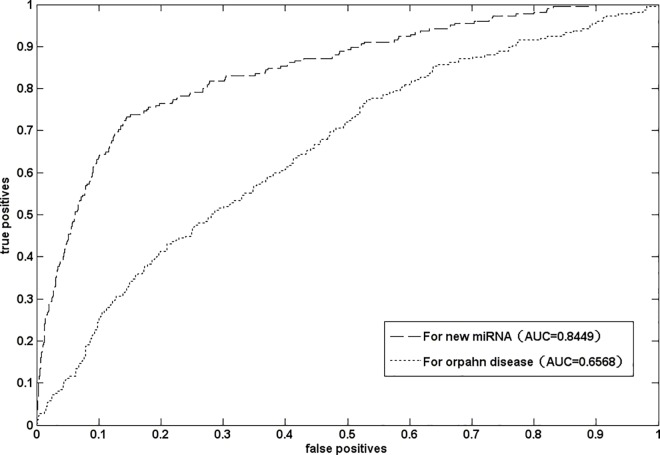
The ROC curve and AUC values of NetGS for new miRNA and orphan disease.

Orphan diseases are a class of diseases which have no related -miRNA information. The new miRNAs are a non-coding RNA for which target disease association information is unavailable. At present, the association prediction for orphan disease and new miRNA is considerably challenging. Many prediction methods can not be applied to solve these two issues. Here, we validated the performance of NetGS for these two issues. To demonstrate that NetGS is applicable to new miRNA, we removed all experimentally verified miRNA-disease associations involved in the query miRNA and conducted the experiment of leave-one out cross validation. The result has shown in Fig 1. NetGS yielded a good AUC value of 0.8449. It indicates our method can be applied to association prediction for new miRNA. To verify that NetGS is able to prioritize miRNAs for orphan disease, all experimentally verified associations related to the query disease were removed. LOOCV was implemented on the gold dataset. The result was shown in Fig 1. As we can see that our method is applicable to association for orphan disease. However, the prediction accuracy still need be improved. The cause of such result is that the similarities of disease pairs are very low. The prediction accuracy may be improved by introducing new disease similarity measurement.

### 4.3 Comparison with other miRNA-disease association prediction methods

**Fig 2:**
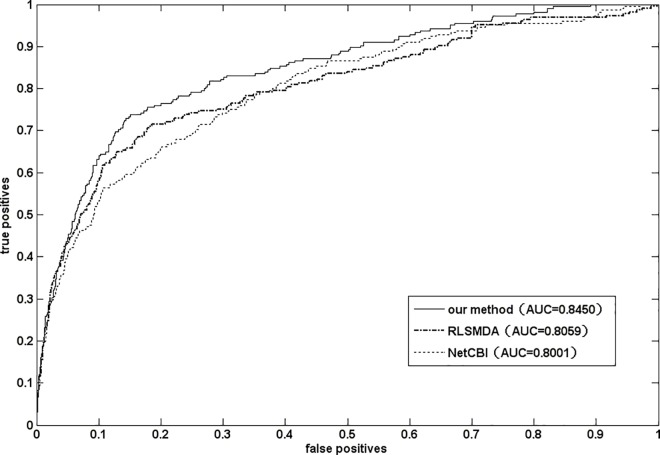
The ROC curves and AUC values of RLSMDA,NetCBI and our method(NetGS)

Until recently, some miRNA-disease association prediction methods have been proposed. As we known, the HMDP [29], RLSMDA [19], NetCBI [18], and the global network algorithm developed by Shi et al. [17] are -state-of-the-art methods. However, HMDP can only be applied to diseases which are associated with at least 60 miRNAs. So, it is not comparable to our method. The method developed by shi et al. [17] integrated the information of disease gene associations, miRNA target interactions, and protein interactions which were totally different from the information used in this paper. As a result, it can’t be compared with our method in a fair way. Here, we compared NetGS with RLSMDA and NetCBI based on the gold standard dataset. The results have been shown in the Fig 2. We can see that NetGS is superior to the other two methods. The good performance may due to the following factor. First, we employed graph Laplacian scores to exploit the global similarity of miRNA and disease. Second, the initial vector of random walk with restart algorithm was optimized by introducing the initial distribution vectors of similar diseases. Third, the idea of diffusion profile consistency was applied here.

**Fig 3:**
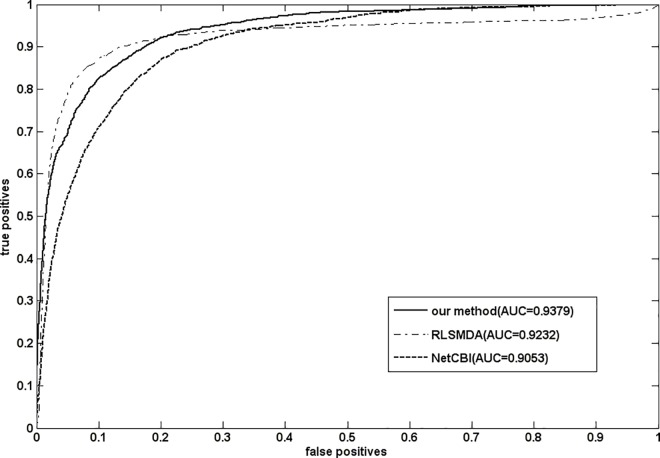
The prediction results of RLSMDA,NetCBI and our method(NetGS) on the second miRNA-disease association dataset.

To validate the prediction result is not sensitive to miRNA-disease association in the gold standard dataset, we conducted LOOCV based on the second miRNA-disease association dataset which had been described in the part of materials. Before experiment, we removed the highly similar associations and the common associations with the gold standard dataset. The prediction results of RLSMDA, NetCBI and our method(NetGS) have been shown in Fig 3. We can see that our method still perform well.

**Fig 4:**
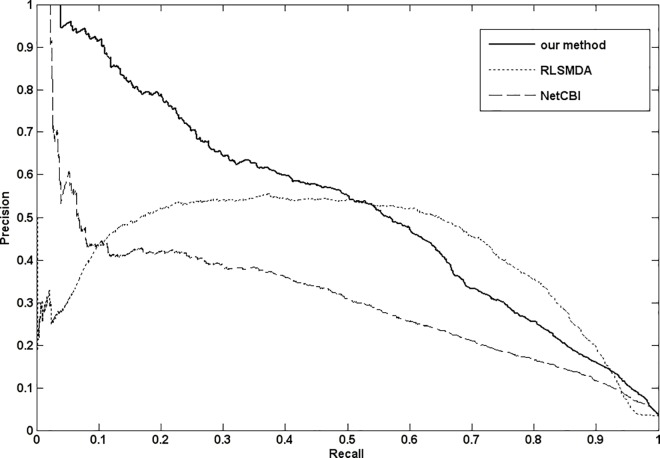
The Precision-recall curves of RLSMDA,NetCBI and our method(NetGS).

At present, another popular evaluation criterion is the precision-recall curve, which includes precision and recall at different thresholds. Precision denotes the proportion of disease miRNAs in all miRNAs, whose prediction association scores are higher than the threshold. Recall denotes the proportion of correctly predicted disease associated-miRNAs among all the disease miRNAs. The prediction results of RLSMDA, NetCBI and our method(NetGS) are shown in Fig 4. We can see that our method still perform better.

### 4.4 The influence of parameters on NetGS performance

In this paper, three kinds of parameters have been used. It includes the restart probability parameter *γ* in the random walk, the weight parameter *λ* in the optimized vector and the balance parameter *α* and *β* in the computation formula of laplacian scores.

**Fig 5:**
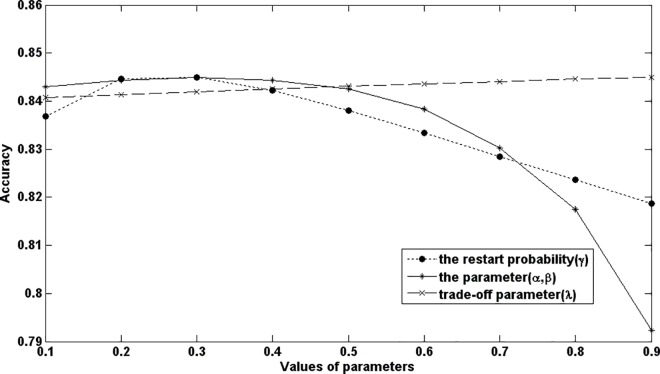
The effect of parameters on the NetGS performance.

The restart probability parameter *γ* controls the probability that the walker return to the source nodes. Here, to test the effect of this parameter, we fixed other parameters and varied the value of *γ* from 0.1 to 0.9 in the LOOCV experiment. The results are shown in the Fig 5. We can see that change of the parameter has little impact on the NetGS performance.

The weight parameter controls the contribution of other disease to the initial vector of the query disease. The balance parameter controls the contribution of the two penalties. We adopt the similar method described in the above paragraph to test these two parameters. The results have been shown in the Fig 5. We can see that the prediction accuracy is robust to these two parameters.

### 4.5 Leave-one-out cross validation in the new validation framework

**Fig 6:**
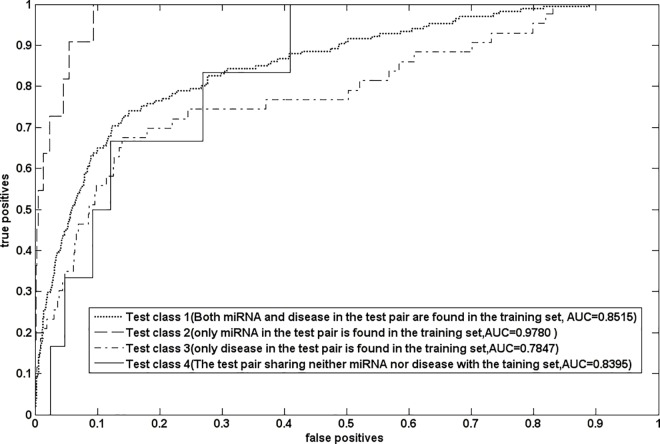
The performance of NetGS in different test classes

Recently, Park and Marcotte have pointed out the flaws in the cross validation for pair-input computational predictions. Their experiment results have been shown that the paired nature of inputs lead to a natural partitioning of the test pairs into distinct classes and the prediction methods achieved distinct performance in distinct classes. According to the validation method they proposed in their article, the test samples were classified into four distinct classes. The test class1 contains the test samples sharing both miRNAs and disease with the training samples. The test class2 contains the test samples sharing only miRNAs with the training samples. The test class3 contains the test samples sharing only diseases with the training samples. The test class4 contains the test samples sharing neither miRNA nor disease with the training samples. The performance of NetGS are shown in Fig 6. We can see that NetGS has achieved reliable prediction accuracies in different test classes.

### 4.6 case study

Substantial evidence indicates that many miRNAs are associated with human cancers and almost half of miRNAs are located in cancer-associated genomic regions or fragile sites. The performance of NetGS have been verified in the above experiments. Here, we used this method to predict causing miRNAs for several harmful and common cancers which are breast cancer, lung cancer and Hepatocellular cancer. We took the known miRNA-disease associations causing miRNA as training samples and the rest miRNAs in the network as the candidate disease miRNAs. Predictive results were confirmed based on the update of HMDD and the datasets in miR2disease and dbDEMC. The top-20 predictions for each disease have been shown in Tables 1–3, respectively.

**Table 1:** The top-20 predicted breast cancer-related miRNA by NetGS based on the gold standard dataset. **Most of them have been confirmed in HMDD** v2.0.

hsa-mir-25	HMDD	hsa-mir-218	HMDD
hsa-mir-1	HMDD	hsa-mir-18a	HMDD
hsa-mir-223	HMDD	hsa-mir-181b	HMDD
hsa-mir-34a	HMDD	hsa-mir-19a	HMDD
hsa-mir-372	unconfirmed	hsa-mir-214	HMDD
hsa-mir-19b	HMDD	hsa-mir-16	HMDD
hsa-mir-133a	HMDD	hsa-mir-92a	HMDD
hsa-mir-143	HMDD	hsa-mir-34b	HMDD
hsa-mir-218	HMDD	hsa-mir-20b	HMDD
hsa-mir-18a	HMDD	hsa-mir-106b	HMDD

**Table 2:** The top-20 predicted lung cancer-related miRNA by NetGS based on the gold standard dataset. 19 of top-20 miRNAs are confirmed in the online datasets.

hsa-mir-155	HMDD	hsa-mir-101	mir2Disease
hsa-mir-19b	HMDD	hsa-mir-146a	mir2Disease
hsa-mir-21	HMDD	hsa-mir-373	HMDD
hsa-mir-92a	HMDD	hsa-mir-214	HMDD
hsa-mir-9	HMDD	hsa-mir-143	HMDD
hsa-mir-451	HMDD	hsa-mir-25	HMDD
hsa-mir-125b	HMDD	hsa-mir-181b	HMDD
hsa-mir-24	HMDD	hsa-mir-20b	uncomfirmed
hsa-mir-145	HMDD	hsa-mir-32	HMDD
hsa-mir-223	HMDD	hsa-mir-16	HMDD

**Table 3:** The top 20 predicted Hepatocellular cancer-related miRNA by NetGS based on the gold standard dataset.

hsa-mir-155	HMDD	hsa-mir-106b	HMDD
hsa-mir-125b	HMDD	hsa-mir-15b	unconfirmed
hsa-mir-15a	HMDD	hsa-mir-101	mir2Disease
hsa-mir-222	HMDD	hsa-mir-451	unconfirmed
hsa-mir-195	mir2Disease	hsa-mir-25	mir2Disease
hsa-mir-20b	unconfirmed	hsa-mir-93	HMDD
hsa-mir-9	HMDD	hsa-mir-214	mir2Disease
hsa-mir-145	HMDD	hsa-mir-29b	HMDD
hsa-mir-126	HMDD	hsa-mir-206	unconfirmed
hsa-mir-106a	dbDEMC	hsa-mir-29a	HMDD

Breast cancer is one of the biggest health killers for woman’s life. According to the report of World Health Organization, the incidence of breast cancer shows a trend of young state. However, at present, the pathogenesis of human breast cancer is still ignorant. As a result, there is a certain blindness to the treatment of breast cancer. Identifying breast cancer-related miRNAs seems very important. Here, in our gold standard dataset, there are 27 causing miRNAs which are known to be related to the development of breast cancer. In this case study, we use our method NetGS and execute the same steps: build miRNA similarity network and disease similarity network, calculate global similarity between breast cancer and potential disease-related miRNA and predict miRNA-disease association scores. After running our method, the top-20 breast cancer-related miRNAs are given in Table 1. We can see that most of the predicted related-miRNAs are confirmed in HMDD v2.0 which validate that the prediction result of our method is reliable.

Lung cancer is one of the greatest threats to human health. It leads to the fastest morbidity and mortality. Also, the pathogenesis of Lung cancer is still ignorant. Identifying related- miRNAs can give a help to the diagnosis, treatment of disease. Here, we took the known lung cancer related-miRNAs as training sample and used NetGS to optimize the candidate miRNAs. The top 20 predicted results are shown in Table 2. We can see that 19 of the top20 miRNAs can be found in the online dataset. The only one which is unconfirmed yet ranks at 18^th^.

The case study about Hepatocellular cancer was conducted in the similar way. The top 20 predicted results are shown in Table 3. Most of them have been confirmed in the online datasets.

## 5 Discussion and Conclusion

MiRNA-disease association inference is the one of the most important goal in biomedical research. In this article, we have presented a novel method to predict miRNA-disease association. This method integrates global information of miRNA similarity network, disease similarity network and the known miRNA-disease associations based on the idea of diffusion profile consistency. As the results show, our method has a reliable performance and has significantly improved other state-of-the-art methods. What’s more, NetGS can solve the new miRNA-disease association prediction problem with reliable accuracy. Case studies about some important disease suggest the practical application of NetGS. In conclusion, NetGS provides a powerful and important tool for disease treatment and drug discovery.

The success of NetGS may be largely lies in the following factors. First, graph Laplacian scores are employed to exploit the global similarity of networks. Second, the initial vector of RWR is optimized. Third, the idea of diffusion profiles consistency is used to compute the miRNA-disease association scores. What’s more, miRNA family information is introduced to measure the similarity of miRNAs. In summary, NetGS represents an important resource for further study.

Despite the promising prediction results of NetGS, some limitations exist in our method. The prediction performance can be improved in the following directions. First, the prediction accuracy of NetGS can be further improved by more available verified miRNA-disease associations. Second, many parameters appear in our model but how to select them is not solved well. Finding better parameter selection methods could improve the performance. Finally, the prediction performance of NetGS for orphan disease-miRNA association is not well. We will make effort to find reliable disease similarity metrics and consider integrating more information to improve the performance of NetGS. Besides, in the future, we intend to apply this method to solve other similar problems, such as lncRNA-disease association, drug-target prediction, and so on.

## Supporting Information

S1 File The minimal underlying data necessary for replication. (RAR)

S2 File The breast cancer related miRNAs functional similarities, miRNA family information, similarity score, constructed miRNA network. (XLSX)
